# Effects of Δ9-Tetrahydrocannibinol (THC) on Obesity at Different Stages of Life: A Literature Review

**DOI:** 10.3390/ijerph19063174

**Published:** 2022-03-08

**Authors:** Nathan Fearby, Samantha Penman, Panayotis Thanos

**Affiliations:** 1 Department of Biological Sciences, University at Buffalo, Buffalo, NY 14203, USA; nlfearby@buffalo.edu; 2 Behavioral Neuropharmacology and Neuroimaging Laboratory on Addictions, Department of Pharmacology and Toxicology, Clinical Research Institute on Addictions, Jacobs School of Medicine and Biosciences, University at Buffalo, Buffalo, NY 14203, USA; slpenman@buffalo.edu; 3 Department of Psychology, University at Buffalo, Buffalo, NY 14203, USA

**Keywords:** Δ9-tetrahydrocannibinol, cannabidiol, prenatal administration, obesity, epidemiology

## Abstract

The *Cannabis sativa* plant has historically been used for both recreational and medical purposes. With the recent surge in recreational use of cannabis among adolescents and adults in particular, there is an increased obligation to determine the short- and long-term effects that consuming this plant may have on several aspects of the human psyche and body. The goal of this article was to examine the negative effects of obesity, and how the use of Δ9-tetrahydrocannibinol (THC) or cannabidiol (CBD) can impact rates of this global pandemic at different timepoints of life. Conflicting studies have been reported between adult and adolescents, as there are reports of THC use leading to increased weight due to elevated appetite and consumption of food, while others observed a decrease in overall body weight due to the regulation of omega-6/omega-3 endocannabinoid precursors and a decrease in energy expenditure. Studies supported a positive correlation between prenatal cannabis use and obesity rates in the children as they matured. The data did not indicate a direct connection between prenatal THC levels in cannabis and obesity rates, but that this development may occur due to prenatal THC consumption leading to low birthweight, and subsequent obesity. There are few studies using animal models that directly measure the effects that prenatal THC administration on obesity risks among offspring. Thus, this is a critical area for future studies using a developmental framework to examine potential changes in risk across development.

## 1. Introduction

*Cannabis sativa*, known commonly as cannabis, is an increasingly popular plant that has been consumed for medicinal purposes for thousands of years, dating back to ancient China. Traditional therapeutic uses of this plant included the treatment of rheumatic pain, intestinal constipation, disorders of the female reproductive system, malaria, and others [1]. Presently, certain phytocannabinoids found within the plant are used for both recreational and medicinal purposes. In modern medicine, some phytocannabinoids have attracted increased attention due to the potential therapeutic uses in many diseases that plague our present population. Most medical studies have revolved around the phytocannabinoid known as Δ9-tetrahydrocannibinol (THC), but other components studied recently include cannabidiol (CBD), cannabigerol (CBG), Δ9-tetrahydrocannabivarin (Δ9-THCV), and cannabidivarin (CBDV) [2].

Throughout the years, the perceived dangers of cannabis have continuously decreased among the adult population, thus leading to certain misconceptions by the public surrounding this drug [3]. Understanding the effects of the consumption of THC is very important, even among adults, due to the fact that this drug is being consumed at high rates. Just within the United States, more than 29.7 million Americans aged 18 years or older reported cannabis use in the past 30 days; this number increased from 2018, when about 26 million Americans in the same age group reported use within this time period [4]. Adolescent use has also been on the incline with about 1.6 million children within the ages of 12 to 17 reporting cannabis use within the last month in 2018; this number further increased to 1.8 million within the same age group and allotted time in 2019 [4]. With further legalization, use among the youth will only continue to rise [5]. These statistics are alarming due to the known effect that THC consumption can have on decreasing the amount of synaptic connections within the developing brain of an adolescent [6].

While there are many potential therapeutic effects of phytocannabinoids in medicine, there are still many concerns and questions. This review will focus on literature on THC, and obesity risks, and include prenatal THC exposure and offspring obesity risk. Additionally, this review will examine the literature on CBD and the physiological responses associated with obesity risks.

## 2. Background and Epidemiology of Obesity

### 2.1. Metabolic Pathways to Obesity

Many factors contribute to what is considered a global obesity pandemic. In 2005, 33% of the global population was considered either overweight or obese. Provided these levels are maintained, it is predicted that by 2030, 57.8% of the world’s population will be considered either overweight or obese [7].

Obesity involves the detrimental interactions between environmental factors that promote weight gain and control systems that contribute to energy homeostasis [8]. The hypothalamus is a critical brain region that controls feeding and energy expenditure. Within the hypothalamus, the arcuate nucleus (ARC) in particular is responsible for the regulation of feeding and metabolism [9]. The ARC consists of two different antagonistic types of neurons that regulate feeding stimulation, known as the anorexigenic (appetite-suppressing neurons) and orexigenic (appetite-stimulating neurons). Extensively studied hormones such as leptin, ghrelin, and insulin mediate expression of orexigenic neurons, including neuropeptide Y (NPY) and agouti-related peptide (AgRP); and anorexigenic neurons, including proopiomelanocortin (POMC) [10,11]. Both POMC and AgRP/NPY neurons project to the second-order neurons of, most importantly, the paraventricular hypothalamic nucleus (PVN) and the lateral hypothalamic area (LHA), but also the dorsomedial hypothalamus (DMH) and the ventromedial hypothalamus (VMH) [12,13]. These neurons found in the ARC have been extensively researched regarding their effects on different pathway activations, and how the development of obesity and hormone regulation can be related. For example, within the AgRP neurons, constitutive activation of the proinflammatory c-Jun N-terminal kinase 1 (JNK1) pathway has been shown to cause increased spontaneous firing within the AgRP neurons, along with systemic leptin resistance. The overactivation of this JNK1 pathway and resistance of leptin both have the effect of stimulating appetite, causing hyperphagia (overeating), increased weight gain, and adiposity [14]. The hormones leptin, insulin, and ghrelin act in different ways in order to mediate the pathway responsible for appetite. Neuronal deletion of the growth hormone secretagogue receptor (GHS-R), a receptor responsible for mediating ghrelin’s effects on appetite pathways, has resulted in reductions in diet-induced obesity and insulin resistance [15].

In contrast, a lack of certain hormone efficacy can result in similar obesity outcomes. Leptin and insulin resistance has been traced back to an intracellular signaling pathway in which leptin is responsible for activation. Leptin-stimulated overactivation of the signaling transducer and activator of transcription 3 (STAT3) and suppressor of cytokine signaling 3 (SOCS3) in POMC neurons resulted in a decrease in energy expenditure, and in turn an increase in body weight, due to the dysregulation of energy homeostasis [16,17]. This resistance of insulin and leptin is thought to be a result of hypothalamic inflammation and increased expression of cytokines due to a high-fat diet [18,19]. In a population of mice with diet-induced obesity, toll-like receptor 4 (TLR-4), which expresses inflammation through the production of cytokines, has been observed to activate nuclear factor-κβ (NF-κβ), leading to a disruption of signaling to the satiety hormone, leptin, and insulin in the hypothalamus [20]. Other studies have also pointed toward other hypothalamic inflammatory markers, such as TNF, interleukin-1, and interleukin-6, which have been seen to significantly increase when rats were fed on a high-fat diet [21]. Thus, the search for possible therapeutic routes in order to control inflammation leading to cell apoptosis is constantly being pursued as a means to maintain energy homeostasis and hormone regulation.

### 2.2. Epidemiology of Obesity

With obesity becoming a more prevalent problem throughout the world, there are major health risks and diseases to which it can often be linked, such as Alzheimer’s disease, dementia, type 2 diabetes mellitus, and coronary heart disease [22,23]. Studies suggest that by 2050, the human population 60 years and older will have increased by 1.25 billion, leading to a prevalence of dementia that will rise to 106 million cases worldwide [24]. Alzheimer’s disease (AD) has been identified as the most common form of dementia, with amyloid beta (Aβ) plaques and tau tangles forming years before the cognitive decline is noted [25]. Enhanced production of Aβ peptides has been investigated as a cause of certain early-onset forms of AD, which led to the amyloid cascade hypothesis—that amyloidogenic Aβ is a very important contributing factor toward the AD pathogenic process [26,27]. Obesity’s health risks with AD is an intriguing correlation, and is known as the obesity paradox [23]. While late-life obesity has been associated with cortical thinning, rapid weight loss has been found to be the leading factor for the elderly in developing AD in weight cohorts [28]. Studies have shown that obesity, measured in the form of body mass index (BMI), in midlife can indeed be a risk factor for AD and developing dementia. In fact, having a BMI corresponding to obesity in midlife is associated with a 3.08-fold increased risk for women and a 2.45-fold increased risk for men for AD, and a 33% increase in vascular dementia prevalence later in life [29]. Rodent models have shown that there is an opposite sex-dependent difference that is suggested in obesity, adiposity, and insulin cohorts; these factors showed that male mice with obesity were more likely than females to have problems with metabolic homeostasis and deficits in learning and memory [27]. Possible therapeutic routes that have been previously investigated involve the leptin pathway, due to its predisposition to cause obesity [16,17].

Type 2 diabetes mellitus (T2DM) is a health issue that has become prevalent, and is connected with and points to the severity that obesity poses as a threat to society. In fact, about 366 million people had developed T2DM as of 2014, and this number has been projected rise to around 552 million people in the next 20 years [30]. It is widely accepted among most researchers that within blood flow and adipose tissue, a decrease in β-cells can result in insulin receptor inhibition, thus leading to a decrease in signaling that would normally avoid cell apoptosis, pointing to a plausible reason for the development of T2DM [31,32,33]. It is still commonly mistaken that obesity is the main culprit in its link with diseases such as hypertension and T2DM. In reality, the largest influence in the development in many of these diseases is metabolic health. Not all individuals with obesity display these metabolic and cardiovascular risk factors, which can describe individuals that have metabolically healthy obesity (MHO). MHO describes the absence of cardiometabolic disease and of the metabolic syndrome components in an individual with a BMI > 30 kg/m [34,35]. MHO individuals have shown that they still have around a four times greater risk of developing T2DM, in comparison to an adult with a normal body mass index (BMI), while metabolically unhealthy subjects had a risk two times that of MHO individuals [36]. However, while ample research has shown that MHO individuals may not be at a higher risk for serious diseases such as hypertension, T2DM, and metabolic syndrome (MetS), other studies have countered this claim [34]. Many different cohorts using body parameters such as body mass index (BMI), waist circumference, and waist/hip ratio have been used in order to predict T2DM and metabolic syndrome. Some studies showed that diabetes was associated with central obesity, in which the adipose tissue was stored such that, as waist circumference and waist/hip ratio increased, so did the chances of developing T2DM [37]. This adipose tissue, an endocrine organ, is associated with many circulating proinflammatory cytokines, such as interleukin-6, which play a part in the development of T2DM when overstimulated [23]. Other studies have shown that BMI, an indicator of total body fat, also is a significant predictor of diabetes, but phenotypes of metabolically unhealthy individuals have a much larger effect, being around 3–9 times that of metabolically healthy individuals [38]. Knowing that inflammation occurs as an effect of obesity, this can point to a link that occurs within the hypothalamus concerning inflammation and T2DM [39,40]. Inflammation from obesity has been linked to the inhibition of the insulin receptor signaling cascade (JNK–IKK–PKR), and can target insulin receptor substrate 1 (IRS-1), causing serine phosphorylation and degradation [41]. Inhibition of this signaling cascade from insulin resistance is associated with endothelial dysfunction, being a factor in the prevalence of developing hyperglycemia and the initial steps in atherogenesis [23,40]. Lastly, insulin receptors are critical to memory, for both plasticity and spatial learning. High levels of insulin receptors reside in the hippocampus, which could suggest a possible role of regulation that insulin has in memory and learning [42]. A lack of insulin receptors has been shown to cause reduced function in the brain, and in particular, impaired hippocampal plasticity and hippocampal-dependent spatial learning [43,44].

Heart conditions are the current leading cause of death in our society. In fact, ischemic heart disease and strokes were attributed to around 13 million deaths globally in 2010 [45]. Obesity is known to be a major risk factor for many different heart conditions and diseases, including coronary heart disease, heart failure, and atherosclerosis [23,46,47]. While obesity does indeed directly affect rates of many of these diseases, conditions that obesity also increases, such as diabetes, high blood pressure, high cholesterol, and metabolic syndrome, are highly effective in the development of many different heart problems [48]. In particular, hypertension has been observed to increase the chances of developing many different heart conditions, and was directly linked to obesity. Studies have shown that body mass index (BMI) had a positive relationship with blood pressure and total cholesterol levels [46,49]. In a study focusing on many different risk factors for developing hypertension, it was reported that waist circumference was the strongest independent predictor (including age, gender, BMI, and insulin resistance) of systolic blood pressure and diastolic blood pressure, accounting for >20% of its variance [50]. Microvascular dysfunction, a disease resulting in reductions in capillary density and arteriolar diameter, has been pointed out as a large contributor to not only obesity, but also hypertension [30,51]. This is thought to be due to the increase in peripheral resistance that subsequently results from the reductions in capillary density and arteriole diameter [30]. The development of more directly life-threatening conditions, such as heart failure, are consistent with their links to obesity. Studies have demonstrated that around 85% of patients having heart failure with preserved ejection fraction, or diastolic heart failure, belonged to populations that were obese; while on the other hand, less than 50% of patients having heart failure with reduced ejection fraction, or systolic heart failure, were considered as obese [47,52]. Meanwhile, other sources disagreed with these findings, and stated that in cardiovascular diseases, mostly coronary heart disease, type 2 diabetes is the most notable risk factor, while also suggesting that obesity was responsible for the inaccuracy seen in a U-shaped curve between BMI and mortality [23]. In general, it cannot be denied that weight most certainly has an effect on the risk of developing many conditions and diseases that involve the heart.

## 3. Cannabinoids and Their Mechanism of Action

### 3.1. The Endocannabinoid System

The endocannabinoid system (ECS) is characterized by four endocannabinoids known as the largely studied N-arachidonoylethanolamine (AEA) and 2-arachidonoyl glycerol (2-AG), and the lesser-studied virodhamine and 2-arachidonyl glyceryl ether noladin, an analogue of 2-AG; these molecules are ligands of two cannabinoid receptors, known as the CB1 receptors (CB1Rs) and CB2 receptors (CB2Rs) [53,54,55]. AEA and 2-AG were given the name “endocannabinoid” due to the nature of these molecules; within the body, these compounds act as endogenous lipids that act upon cannabinoid receptors [54]. AEA is synthesized by the enzyme N-arachidonoyl-phosphatidylethanolamine (NAPE) from arachidonic acid, while 2-AG is synthesized by the enzyme diacylglycerol lipase (DGL), which is stimulated after increased postsynaptic intracellular Ca^2+^ levels or increased activity of phospholipase Cβ (PLCβ) [56,57,58].

Endocannabinoids have been characterized to bind to CB1Rs and CB2Rs, which are G-protein-coupled receptors found within both the peripheral and central nervous systems [54,55,56,58,59]. Although both are found within these systems, CB1Rs are found in high densities within the basal ganglia, substantia nigra, globus pallidus, cerebellum, and hippocampus, about 10–50 fold above those of classical transmitters such as dopamine or opioid receptors, making the CB1R an interesting case to study within the brain [54,56]. The two major endocannabinoids, AEA and 2-AG, are also different with respect to their binding affinities to the cannabinoid receptors. AEA binds to both CB1 and CB2 receptors, but has a much higher affinity for binding to CB1Rs; in fact, the activity of anandamide at CB1Rs is roughly 4–30-fold higher than at CB2Rs [55,56,59]. The 2-AG endocannabinoid appears to bind to both CB1 and CB2 receptors with the same affinity, but at a lower level at both receptors with respect to AEA [55,60].

The process of synaptic transmission throughout the endocannabinoid system is unique in the sense that it acts as a retrograde synaptic messenger. Endocannabinoids are not stored within vesicles in the presynaptic membrane like typical neurotransmitters are; rather, they are synthesized when and where they are needed in the postsynaptic membrane, and bind to the cannabinoid receptors that are located in the presynaptic membrane [54,55]. Historically, the ECS has been linked to many different physiological and behavioral processes. The ECS has a few contrasting physiological roles, mainly consisting of a modulator in GABAergic and glutamatergic synapses throughout synaptic transmission, a regulator of neurotransmitter release within the presynaptic membrane, and induction of long-term synaptic plasticity leading to long-term potentiation (LTP) and long-term depression (LDP) [54,55,56,58,59]. Experimental data have shown that this decrease in inhibitory GABAergic and glutamatergic synaptic transmission was due to a G_i/o_ protein-mediated modulation of K^+^, but not of Ca^2+^, conductance [61]. Activation of the CB1Rs by AEA or 2-AG within the presynaptic terminals has also been documented to suppress the release of cholinergic, serotonergic, and noradrenergic neurotransmitters throughout the many different spots within the central nervous system (CNS) [58]. This lack of neurotransmitters can result in the excitatory postsynaptic potentials (EPSPs) of the terminal synapses, either increasing or decreasing depending on the activation of the CB1R, which can result in long-term plasticity, such as LTP and LDP [59,61].

Changes in ECS signaling have demonstrated effects on behavior, most notably occurring within the amygdala [62]. Keeping this in mind, the ECS is found throughout the brain and within neuronal circuits that control certain behaviors such as anxiety, fear, stress, and addiction [58,62]. For example, one of the most heavily studied of these behaviors, anxiety, is directly influenced by cannabinoid levels in a biphasic manner, with low doses contributing to a anxiolytic state, and high doses contributing to a anxiogenic state [58,63]. Some studies present these same results as a decrease in exploratory behavior in conjunction with lower levels of CB1R activity [64]. Important cognitive processes such as learning and memory have also been linked to the ECS. Certain studies have tested rodent models by administrating CB1 receptor agonists and inducing the animals to perform various memory tasks, including the Morris water maze, novel object recognition, and contextual fear conditioning; these results showed that CB1 receptor agonists attenuated the acquisition of memory, and therefore increased levels of AEA and 2-AG can elicit these same results [63,65,66,67]. There are inconsistent data that the endocannabinoid degradation factors fatty acid amide hydrolase (FAAH) and monoacylglycerol lipase (MAGL) are inhibited during an increase in memory retention [68,69]. FAAH is mainly responsible for the degradation of the endocannabinoid AEA, and its shorter, monosaturated N-acylethanolamine (NAE) analogue, oleoylethanolamide (OEA), into arachidonic acid and oleic acid, respectively [54,55,56,70]. In contrast, MAGL with FAAH (to a lesser degree) hydrolyzes 2-AG back into arachidonic acid [54,55,56,70]. Inhibition, or genetic deletion, of these degrading factors has been shown to increase endocannabinoid levels, due to more neurotransmitters being available to bind to CB1 and CB2 receptors [58,70].

This increase in endocannabinoid levels also is a modulator of inflammatory pain [71,72]. Inflammatory pain paradigms have pointed to fatty acid binding proteins (FABPs) as a means to clinically produce antinociceptive effects [73,74]. FAPBs in the endocannabinoid system, mainly FABP3, 5, and 7, act as chaperones for the transportation of endocannabinoids to their degrading factors [73,75]. Therefore, by inhibiting, or genetically deleting, these FABPs, increased levels of AEA, 2-AG, and other NAEs have been recorded in order to produce the antinociceptive effects that are modulated within the ECS [73,74,75].

### 3.2. Phytocannabinoids and Synthetic Cannabinoids

Within the *Cannabis sativa* plant, there are over 500 compounds, including 104 cannabinoid-type compounds, that play different physiological roles inside the ECS, as well as other systems throughout the body [2,76]. These compounds are known as phytocannabinoids, as they are the natural cannabinoid components from the plant that interacts with cannabinoid receptors, or in the case of noncannabinoid components of the plant, share chemical similarities with cannabinoids or can act as a cannabinoid receptor ligands [77]. Among the 113 phytocannabinoids [78], these compounds are separated into 11 distinct classes that include: (−)-Δ9-trans-tetrahydrocannabinol (Δ9-THC), (−)-Δ8-trans-tetrahydrocannabinol (Δ8-THC), cannabinol (CBN), cannabidiol (CBD), cannabitriol (CBT), cannabinodiol (CBND), cannabielsoin (CBE), cannabicyclol (CBL), cannabigerol (CBG), cannabichromene (CBC), and miscellaneous cannabinoids [76,79]. Of all of these cannabinoid types, the major phytocannabinoids are cannabinol (CBN), cannabidiol (CBD), cannabigerol (CBG), and (−)-Δ9-trans-tetrahydrocannabinol (Δ9-THC). CBN was the first cannabinoid to be isolated from *Cannabis sativa,* at the end of the 19th century, and has hallucinatory activity, although at a much lower level than Δ9-THC [79,80]. The second cannabinoid to be isolated was CBD, which in contrast has no such hallucinatory activity [2,79,80]. Δ9-THC was then discovered after the synthetic activity of Δ6a,10a-tetrahydrocannabinol was similar to that of the natural phytocannabinoid [79]. Δ9-THC has the strongest hallucinatory effects of all other phytocannabinoids, with 11-OH-THC being the main psychoactive metabolite of Δ9-THC [80]. Δ9-tetrahydrocannabolic acid A (Δ9-THCA) is the direct acidic precursor [81], and is derived through either heating the plant up or through storage and fermentation, which causes decarboxylation slowly over time [81]. Both CBD and Δ9-THC are derived from the precursor CBG, and the major homologues cannabidiverin (CBDV) and Δ9-tetrahydrocannabivarin (Δ9-THCV) derive from the major homologue cannabigerovarin (CBGV) [2].

Synthetic cannabinoids have also been rapidly emerging as popular drugs of abuse, widely known as “K2”, due to the intense potency, high cannabinoid receptor affinity, and the lack of activity shown on drug screenings [82]. There are currently more than 20 different synthetic cannabinoids that are identified and bind to cannabinoid receptors, causing known effects such as hypothermia, analgesia, and catalepsy [83]. There are many different types of synthetic cannabinoids that affect contrasting parts of the endocannabinoid system. In particular, there are five major categories of synthetic cannabinoids: classical cannabinoids, nonclassical cannabinoids, eicosanoids, hybrid cannabinoids, and aminoalkylindoles [55]. These synthetic cannabinoids can have an effect by acting as agonists and antagonists for degrading enzymes, CB1/CB2 receptors, and other non-CB1/CB2 receptors [70]. In reality, though they may act in a similar manner to endocannabinoids and synthetic cannabinoids, drugs carrying synthetic cannabinoids are highly toxic and dangerous, not only due to the high CB1 receptor stimulation that they elicit, but also the unknown contaminants that are within the drug [83].

## 4. Δ9-Tetrahydrocannabinol (THC)

The primary psychoactive compound of *Cannabis sativa*, Δ9-tetrahydrocannabinol (THC), has been extensively studied for its effects across development and health. The structure of THC was first discovered in 1964, with synthetic creation of this drug following soon after [54]. Although the effects of THC on the brain are generally known, there are still many factors that have been extensively studied that conflict with each other.

Much of THC research revolves around the effects that the compound has on different parts of the brain containing high densities of CB1Rs, such as the amygdala, frontal and prefrontal cortices, cerebellum, and hippocampus [54,84]. Most studies have focused on THC effects within the prefrontal cortex and hippocampus, and specifically, changes in cognitive and executive functioning, learning, memory, and attention [54,85]. Results of human studies have shown that chronic use of THC resulted in impaired cognitive and executive functions, including short-term memory, verbal episodic memory, attention, and learning capabilities [84,86,87,88]. This has been demonstrated in numerous rodent models as well [89,90,91].

The opposite effects have also been shown [92] with acute and chronic (1.5 mg/kg) THC, inducing a high discriminatory index (rats spent less time focusing on the familiar object in comparison to the control rats). Male and female rats were also found to have their working memories enhanced by acute THC [93]. Along with this, and the fact that BDNF and other hippocampus proteins exhibited a higher expression within the THC-treated rats in both [92,94], it was concluded that low-dose acute and chronic THC administration resulted in increased memory and learning processes. Other aspects, including decision making mediated by the amygdala and orbitofrontal cortex, have also been tested as a factors affected by THC, but at a much lower level than in the studies that explored cognitive and executive functions. Human studies, such as [95], have demonstrated poor daily decision-making processes by chronic cannabis users in comparison to nonusers.

Routes of administrations have also been seen to result in differences in blood and serum THC levels when using pharmacokinetic studies. Historically, the use of oral, injected, or smoked methods have been chiefly studied in order to determine the THC metabolite presence within the body. Oral administration resulted in cannabinoid levels not elevating until 4–6 h after ingestion due to slow absorption, and the metabolite peaks and overall bioavailability were lower [96]. Injection resulted in peak levels of THC within the plasma around 10 min after administration, and showed high levels of THC and 11-OH-THC in the plasma and brain [96,97]. Smoking typically had the fastest peak increase in cannabinoid presence within the plasma, but typically showed lower metabolite levels than those of an injected administration [96,97]. However, due to the large increase in popularity of e-cigarette usage [98,99], vapor administration has recently become a heavily researched route of administration. Interestingly, an animal model concluded that vaporized inhalation resulted in higher levels of THC within the plasma and brain, while injections resulted in higher levels of 11-OH-THC [100]. Overall, further research should be done in the future in order to be able to mitigate the differences between THC concentrations in animal models and humans based on different routes of administration.

It is a widely held misconception that cannabis use within the adult population has no serious consequences due to the brain having finished developing. While this may be partially true, there is still much not known regarding both the short- and long-term consequences of THC and its dosing, but the data seem to vary regarding cognitive and executive function, learning, memory, verbal recall, and even social behavior. Much of the literature published on THC use and its effects on cognitive function seems to vary greatly. In particular, there were human studies suggesting that midlife cannabis use, even when used chronically, had no effects on verbal recall and other cognitive functions [87]; while in other cases, rodent models that went as far as to propose that THC at low doses had the ability to restore the cognitive performance of advanced-age mice back to the hippocampal gene expression levels of 2-month-old mice (considered young) [94]. Chronic THC use in rodent and human studies, and given shortly before cognitive tests (verbal learning tests and recognition recall), resulted in decreased cognitive performance and decreased activity in brain regions involved in memory and attention, as well as synaptic changes and reduced volume within the hippocampus, prefrontal cortex, and cerebellum [86,101,102,103,104]. These same findings were supported in rodent models [91,105,106] in studies that used the Morris water maze/spatial memory and novel object recognition to test cognitive function). When THC was administered just before memory or learning tasks, regardless of dose, this decline in performance was observed. However, there is debate over the longer-term effects of THC after administration, especially when delivered chronically. Both human and rodent studies have concluded that chronic administration of THC resulted in long-lasting effects, including on brain structure and behavior, especially within the adolescent brain [91,101,107,108,109]. Others have found the opposite: that chronic exposure to THC had no long-lasting effects on the neuroplasticity and connectivity within the nervous system [94,105,106]. Of course, these differences across studies and models could be due to differences in amount or potency of THC, developmental period (even within adulthood), measurement and route of administration.

## 5. Effects on Obesity across Development: Δ9-Tetrahydrocannabinol (THC)

**Figure 1:**
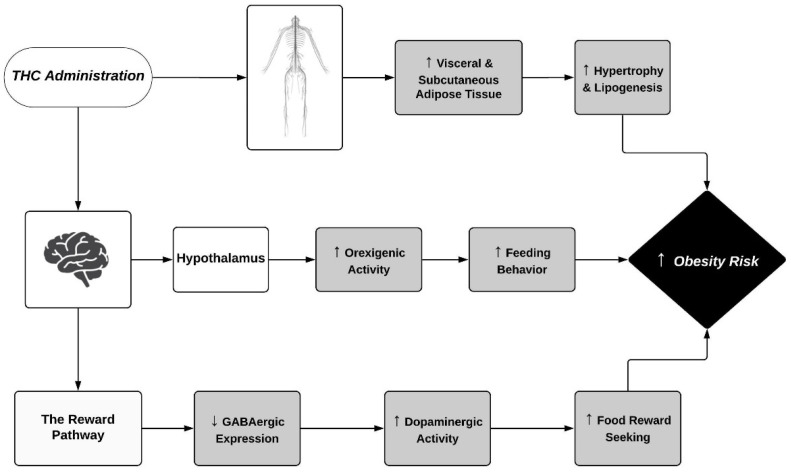
This mechanisms by which THC administration affects the physiological responses to obesity risks through both the central and peripheral nervous systems. The Central nervous system processes include the orexigenic pathway through the hypothalamus and the reward pathway through the nucleus accumbens and ventral tegmental area. Peripheral nervous system processes largely include the retention of visceral and subcutaneous adipose tissue. Symbols ↑ and ↓ denote an observed increase and decrease, respectively.

The CB1Rs, through which THC’s mechanism of action occurs, also seem to have a large role in obesity risks due to high densities within the hypothalamic and reward circuits (Figure 1), which regulate feeding behavior, regulation of intake, and satiety [110,111,112,113,114,115]. CB1Rs that are intact, bound to, and activated via endocannabinoids or phytocannabinoids displayed an increase in food seeking and body weight gain when mice were fed a high-fat diet [111]. The hypothalamus is responsible for not only integrating both the central and peripheral feeding signals, but also regulating energy intake through pathways that stimulate hormones, such as leptin and ghrelin, in order to trigger orexigenic and anorexigenic mechanisms (Figure 1) [111,113]. Although the hypothalamus does not have as high of a density of CB1Rs as other regions within the brain, G-protein activation seems to be especially efficient in this area [116]. However, subregions within the hypothalamus, such as the PVN, have much higher quantities of CB1Rs that play a large physiological role in modulating neurons involved in feeding [117]. Specifically, the PVN is considered second-order hypothalamic neurons responsible for stimulating orexigenic pathways, thus activation of the CB1R by a partial agonist such as THC cannot only have large modulatory effects on the function of such cells, but also increase feeding motivation [12]. As for the reward system, the mesolimbic dopamine pathway, which involves the ventral tegmental area (VTA) and the nucleus accumbens (NAc), promotes feeding behavior in order to induce the pleasant sensations of obtaining the natural reward, being food, while simultaneously inducing reinforcement signaling (Figure 1) [112,113]. Within the VTA, there are high levels of dopaminergic neurons that are responsible for the reward behaviors associated with increased feeding [118]. These dopaminergic axons target GABAergic neurons that are responsible for the reduced expression of dopaminergic neurons through neurotransmitter release, which in turn are inhibited by the activation of the presynaptic CB1Rs through increased levels of THC within the brain, therefore exhibiting high reward-seeking activity through increased expression of the dopaminergic neurons [118].

Many studies have examined this link between CB1Rs and feeding regulation by removing these receptors in the hypothalamic and reward circuits. In general, CB1R knockout mice maintained on standard diets remained lean and were resistant to developing obesity, even when put on high-fat diets [111,114,119]. THC, a partial agonist of the CB1Rs, has therefore been extensively studied regarding its effect on energy intake and feeding behaviors by modulating these different pathways. Major findings using rodent models showed THC was responsible for inducing hyperphagia through increased energy intake and feeding behaviors [112,120,121], with others suggesting an increase in sucrose intake, or liking sweets, due to a THC-induced palatability shift [111,121,122,123]. These data were further confirmed through the use of CB1R antagonists such as SR141716 and AM251. Blocking the CB1Rs had the effect of reversing the outcomes caused by the partial agonist, THC, through decreased appetite, palatability, caloric intake, and body weight [124,125,126,127].

Due to the high levels of co-use of cannabis (THC) with different drugs, chiefly alcohol and nicotine [98,128], how the co-exposure to these drugs may affect obesity outcomes among individuals should also be taken into consideration. Cohorts containing adults that were simultaneously exposed to cannabis and tobacco were found to have pharmacokinetically decreased THC serum concentrations [129]. These effects that nicotine had on THC concentrations could potentially work to decrease obesity rates when used comorbidly, especially when accounting for nicotine’s characteristics as a stimulant. Differently, alcohol was observed to have a positive outcome on plasma THC levels due to the increase in metabolite levels as well as the absorption rate due to co-exposure [130], thus potentially raising obesity risks. However, it should also be noted that in an animal model that identified the metabolic outcomes of alcohol and cannabis co-exposure, the researchers observed a beneficial effect on obesity rates that was explained by improved glucose and insulin homeostasis [131].

THC and its metabolites also seem to have a reasonable effect on physiological processes regarding glucose uptake and metabolism. Certain studies have shown that in the CNS and among peripheral tissues, the CB1Rs seem to impair glucose tolerance and increase insulin resistance, resulting in increased body weight and obesity [131,132,133,134] In particular, one study reported that doses of THC > 1 mg/kg had the effect of decreasing the amount of glucose uptake within certain areas of the brains of rats [116]. This was further confirmed in a study that presented the finding that activation of the mitochondrial membrane CB1Rs hampered the activity of astrocytes, resulting in decreased levels of glucose uptake in the blood–brain barrier [135]. These results can be explained by the CB1Rs being responsible for altering the phosphorylation of the mitochondrial subunit, NDUFS4, therefore resulting in a reduction in reactive oxygen species for the astrocyte in order to produce lactate through the hypoxia-inducible factor 1 pathway [135,136]. The results from these studies were significant, showing that glucose played an imperative role in regulating energy so that physiological processes can occur within functioning cells.

Excess adipose tissue has increasingly become a critical factor in the development of many complications that increase the rates of obesity, such as insulin resistance, glucose intolerance, cardiogenic factors, and metabolic syndrome [137]. CB1Rs were also found to be dense within the periphery, especially within adipose tissue, where increased receptor activation resulted in elevated levels of both subcutaneous and visceral adipose tissues (Figure 1) [132,138]. In [139], a study of chronic cannabis users found a significant increase in abdominal visceral fat, as hence was in line with the findings that cannabis was associated with a larger waist circumference. This was explained by THC exposure being responsible for adipose infiltration and increased hypertrophy and lipogenesis (Figure 1) [140]. A specific model using pigs as the experimental animal found that THC reached fat tissues quickly, and due to its lipophilic properties, had a slow elimination and high retention rate within these fat storages, allowing for increased CB1R expression in these adipose tissues [141]. Nonetheless, further research must be done in order to confirm the effects that the CB1Rs, and in particular the drug THC, have on body weight, metabolism, and behaviors.

The opposite conclusion has also been recorded through human surveys, in which cannabis users were less likely to be considered overweight in comparison to nonusers, even when accounting for the increased appetite that arose due to cannabis consumption [142]. Another human study explained this paradox through THC being responsible for the downregulation of the CB1Rs, thus reducing BMI and obesity levels due to regulation of the ratio of omega-6/omega-3 endocannabinoid precursors [143]. Furthermore, although THC may be responsible for an increase in food intake, it was also associated with reductions in energy intake and reserves [119,144,145]. The authors of [119] reasoned that these results were mainly due to an inhibition of increased fat masses. In addition, it also was stated that THC and its metabolites, which have been reported to produce hyperphagia, had a positive effect on the metabolism of glucose uptake through stimulation of CB1Rs within both centrally and peripherally mediated cells [146]. These findings were thought to be partially due to the role that astrocytes play as glucose transporters within the brain. Among glucose transporters, specifically, GLUT2 is highly expressed within the hypothalamus, which is responsible for control of orexigenic and anorexigenic pathways, and is found on these astrocytes [136]. This may be clinically significant due to findings that THC was responsible for increased glucose uptake via glucose transporters, specifically when plasma levels of THC were similar to those found in actual models of human administration of cannabis [147].

Many of these differences in findings were due to the fact that THC has been regarded as inducing biphasic effects when activating the CB1Rs [55]. Possible explanations of this biphasic effect include the fact that THC acts as a partial agonist at the CB1Rs, and thus at certain doses and conditions acts as an agonist stimulating the effects that other endocannabinoids induce; while at other doses and conditions, it acts as a blocker of the receptor properties [117,148,149]. These effects were not only dose-dependent, but also were demonstrated within different routes of administration. Not only were factors regarding metabolism, induced body weight, and rewards stimulated by THC’s biphasic effects, but also domains such as memory and cognition [148,149].

### 5.1. Effects of Δ9-Tetrahydrocannabinol (THC) on Adults and Obesity

With regard to the effects that THC has on obesity risks, specifically among adults, there seems to be mixed results among the literature within this area of research (Table 1). Among human studies, there were some models that suggested chronic use of THC could be linked to a reduction in the amount of insulin that is secreted, thus resulting in less compensation for the reduced insulin sensitivity. The study in [150] suggested that this could be due to cannabis causing an overexpression of uncoupling protein-2 (UCP-2), which is responsible for the regulation of ATP through control of the number of protons reentering the mitochondrial matrix during the oxidation of substrates [151]. Hence, when overexpressed, they can be used as a therapeutic site of action in order to reduce the buildup of insulin resistance, which is a synonymous complication with obesity [150].

Other survey-style human studies also included the impact that cannabis use had on obesity, as well as insulin resistance. One such study focused on an Inuit population, and found that increased use of cannabis resulted in lower levels of BMI, % fat mass, fasting insulin, and insulin resistance [154]. Another study among US adults found similar results, with increased use of cannabis within the last month resulting in lower levels of fasting insulin and insulin resistance, while also producing a smaller waist circumference [155]. In [155], the authors explained these findings as being due to the adipose tissue, adiponectin, which was activated through the antagonistic effects of the partial agonist THC, and was found to improve insulin sensitivity. In contrast, Ngueta et al. [154] suggested many different mechanisms of action that describes these results from THC administration, such as increase in immune response, differences in adipose tissue metabolism, and increased energy expenditure. Although much time has been spent on both the central and peripheral effects in conjunction with THC, there still seems to be many questions regarding the mechanism of action this drug, specifically regarding uses in order to exert these specific effects.

The effects of THC administration on obesity risks within adults have also shown opposing findings (Table 1). Specifically, Muniyappa et al. [139] demonstrated that chronic, daily cannabis use resulted in an impaired adipose tissue insulin sensitivity, explained by higher levels of THC and prolonged retention in tissues, greater sensitivity to antilipolytic effects of insulin in these tissues, and the amount of CB1Rs being expressed in insulin-sensitive tissues (Figure 1). In [157], the authors went on to observe impaired glucose tolerance, depending on the route of administration. This was further elaborated by a study that associated a higher use of cannabis with an increased prevalence of prediabetes during middle adulthood [156]. The reasoning behind the results demonstrated relied on the route of administration that is the most popular clinically: smoking. Thus, the metabolically favorable effects that occurred from THC use on the anti-inflammatory immune response were countered by the detrimental oxidative effects, due to the production of oxidative species and stress from inhaling smoke into the lungs [156].

Other effects that THC use has shown on obesity risks have also been observed as well. For example, a study regarding a CB1-receptor-mediated modulation of food intake in mice observed an increase in caloric intake when THC was administered, while these effects were reversed through administration of the CB1R antagonist SR141716A [127]. Among human studies, these same effects were observed, as THC acted as a stimulator of appetite, increased food and caloric intake, and increased body weight, and resulted in a larger waist size [152,158]. Clinically, these effects that THC has on increased caloric intake have even been used medically for HIV-positive and other sick patients in order to aid in maintaining body weight [153,159]. In order to explain these results, the authors of [127] showed the mechanism of action to be CB1-mediated within the hypothalamus (Figure 1), thus altering levels of neuropeptides and orexigenic and anorexigenic pathways; the authors of [152] drew similar conclusions. Either way, the fact stands that further research will have to be done in order to completely understand the effects that THC has on metabolism and body weight within the adult population.

### 5.2. Effects of Δ9-Tetrahydrocannabinol (THC) on Adolescents and Obesity

The effects that THC exerts metabolically and behaviorally within adolescent populations appear to be similar to those found in the literature on adults. Much of what did differ between the literature on adolescents and adults regarding the effects THC has on obesity risks was the models of testing (Table 2). Adult research found regarding this topic heavily consisted of human models that used a survey style, while most adolescent studies were in animal models due to ethical aspects. Nevertheless, Farhat et al. [160] measured the relationship present in human studies between cannabis users and body weight. It was displayed that chronic use of cannabis in adolescence resulted in an increased proportion of overweight/obesity risks, with a sex-dependent effect of being more present within the female population. In an observational analysis of feeding patterns induced by THC in rats, it was found that THC acted as an agonist on CB1 receptors and resulted in increased feeding, incentive value of food, and feeding motivation [112]. The mechanism by which the authors explained this increase in appetite and decrease in eating latency was by activation of the mesolimbic dopaminergic pathway, or the reward pathway (Figure 1), and orexigenic pathways stimulating food intake within the hypothalamus [112]. Other studies that included the use of female rats in an activity-based anorexia (ABA) model with constant use of a running wheel also showed a clinical value in attenuating the loss of body weight with the use of a high-fat diet (HFD) [161], and also increased the behavioral effects through stimulation of leptin release and decreased plasma levels of corticosterone [162]. In addition, with increasing feeding behavior, THC also had the effect of reducing the expression of UCP1 genes, which are responsible for thermogenesis within brown adipose tissue, while demonstrating reductions in energy expenditure and lipolysis (ATGL and CGI-58) within white adipose tissue (Figure 1) [161]. Results of studies on how THC interacted within the adolescent age group seemed to show a hyperphagic effect, which also has been examined as a potential therapeutic agent in order to increase the weight of sick patients.

Similar to the data for adults, adolescent THC administration has also resulted in the opposite effect: a decrease in body weight, reduced insulin resistance, and increased energy metabolism and homeostasis (Table 2). A study of how THC altered the body weight of males found that chronic administration resulted in an overall decrease in body weight from the vehicle [163]. Furthermore, in a study that involved mice induced with an HFD, not only did chronic administration of THC prevent diet-induced obesity, but also reduced fat mass gain and energy intake without effecting locomotor activity [119]. This study took another approach and concluded that chronic administration of THC led to modification in the mice gut microbiota, hence leading to a reduction in weight gain induced by an HFD [119]. In [131], the authors also observed that a subcutaneous THC injection also resulted in reduced adiposity; specifically, lowering the amount of visceral fat, along with other positive outcomes, including increased insulin sensitivity and increased glucose homeostasis. At present, the specific effects that THC administration has on obesity risks within adolescents seem to be due to many factors such as dose, regimen of drug administration, and sex. In order to truly understand the relationship between THC and obesity risks, more work must be done.

### 5.3. Effects of Δ9-Tetrahydrocannabinol (THC) on Prenatal/Perinatal and Obesity

Open use of cannabis by women during pregnancy has started to become a large topic of discussion due to the increasing perception that regular cannabis use has little risk. This perception increased threefold from 2005 until 2015, and has only increased more since [164]. Studies have also found that these perceptions played a large role in the increasing trend of cannabis use among pregnant woman [165,166,167,168]. These results can be very shocking when considering the effects that a CB1R agonist such as THC can have on a developing brain. CB1 agonists are critical in many different neuronal functions such as neuronal fate and synaptogenesis, and by indirectly regulating the firing of the CB1Rs on the presynaptic terminals, adverse effects on brain development and synaptic plasticity can occur [6]. Largely, many studies have noted a relationship between prenatal use of cannabis and the detrimental effects it had on the infant’s executive functioning, such as cognitive flexibility, sustained and focused attention, planning and working memory, and even emotionality [169]. Recent articles have found a relationship between co-exposure of tobacco and cannabis and having higher odds of belonging to populations with obesity compared to no exposure [170]; however, there is still room for research to be done on THC’s effects alone. Past research also began to notice a trend within prenatal cannabis use and its outcome on infant size and weight. The birth weight of an infant seemed to have a direct relationship with growth later in life, and even in obesity risks later in life (Table 3).

#### Impact of Low Birthweight on Obesity

One of the overarching goals of this review paper was to highlight the effects of prenatal THC use on obesity risks later in life, and with increasing literature about this topic coming to the surface, there seems to be an interesting trend between the two (Table 3). Specifically, it was observed that cannabis administration during pregnancy can lead to low birth weight or preterm delivery [173,177]. One study that examined the birth outcomes associated with cannabis use before and after pregnancy observed a strong and significant positive relationship between cannabis use and negative birth outcomes such as low birth weight, preterm birth, and admission into a neonatal intensive care unit [174]. Another study also observed the specific deleterious effects of smoking on an infant’s birth weight [172]. Possible mechanisms that the authors used to explain these outcomes included the possibility that prenatal smoking resulted in increased levels of low birth weight due to the vasoconstrictive action of fetal hypoxia. Prenatal stunted growth can likely lead to higher levels of overweight children due to the abnormal postnatal growth patterns induced from smoking [172]. Within mice, similar results were observed, but with the male gender, having an increased susceptibility to this reduced birth weight was the outcome [176]. An experiment that examined this link within pregnant rats found that 3 mg/kg THC administration intraperitoneally resulted in fetal growth restrictions as a result of impaired placental development [171]. These findings were imperative, due to the fact that fetal growth restriction seemed to have a direct relationship with intrauterine growth restriction due to decreased nutrition from the lack of GLUT1, which subsequently led to other diseases, including obesity, type 2 diabetes, and metabolic syndrome [171].

Low birth weight is generally caused by two different factors during pregnancy, the first being preterm birth, and the other being intrauterine growth restriction [178]. Specific mechanisms behind the use of cannabis and a decrease in the embryo development can be explained by the binding of the CB1R agonist, THC, which causes modulatory effects on many growth factors, including vascular endothelial growth factor and proliferating cell nuclear antigen, and pathways involved in modulating gene expression of cell neurogenesis and proliferation [179]. Vascular endothelial growth factor, which is critical in order to stimulate cell neurogenesis within the fetus, has been found to be inhibited through THC administration into a rat C6 glioma cell culture [175]. The impact of decreased expression of proliferating cell nuclear antigen as a result of CB1 agonist administration has also been observed, with a decrease in fetal development due to the lack of cell replication resulting from the decreases in levels of these growth factors [179]. These effects were due to the fact that lipophilic THC rapidly crossed the placenta, and even though blood plasma levels were lower, they were comparable to the maternal blood THC levels [180].

Low birth weight seems to have a strong association with not only obesity risks, but also obesity-related diseases. In [181], the researchers found a significant relationship in which low birth weight was a strong predictor of obesity at the age of 8 years. These results were explained by the rapid weight gain and growth of the infant from the ages of 1 to 7 years due to the concept of “catch-up growth” [181]. The low weight at birth caused a physiological response by the body in order to stay on pace with a normal weight, and this rapid weight gain during this time period is what caused the elevated obesity risks later in life. This study even went as far as to conclude that the amount of weight gain from ages 1 to 7 years could be a predictor of these same risks for these individuals, even into their 40s [181]. Similar results were seen in increased prenatal smoking [172] and cannabis administration [173]. Other studies observed these same results, but also with elevated risks for obesity-related diseases. In many population-based studies, low birth weight also resulted in an increase in obesity-related problems such as leptin resistance due to the elevated hormone levels from the catch-up growth phase, T2DM, high blood pressure, cardiovascular disease, and insulin resistance [178,182,183,184,185]. Nevertheless, the effects that low birth weight seems to have on potential health outcomes later in life should create an increased level of concern, due not only to THC’s direct effect on an infant’s birth weight, but also to the growing perception that cannabis use has no risk involved.

## 6. Cannabidiol

*Cannabis sativa* contains several different phytocannabinoids that have an impact on the endocannabinoid system and result in many different physiological responses. In addition to THC, another popular phytocannabinoid known as cannabidiol (CBD), has also been getting a large amount of attention due to its therapeutic relevancy in pain modulation and its anxiolytic properties. Along with this, CBD has also been demonstrated to attenuate many of the negative effects that are produced by THC administration, such as memory impairments and increased obesity risks (Table 4) [54,115]. Understanding the role that CBD has in the endocannabinoid system and how it can overall impact certain functions within the body can allow for possible therapeutic mechanisms for increasingly concerning healthcare problems, such as obesity.

As stated, when only CBD was administered, there were several therapeutic characteristics, including anxiolytic, antidepressant, antipsychotic, and anti-inflammatory [195]. CBD can achieve these affects through multiple receptor-independent pathways. Within the cannabinoid receptors, CBD is a partial agonist at the cannabinoid 2 receptors (CB2Rs) found within the CNS and immune system cells, which also have a low affinity for CB1Rs [196]. In fact, CBD tended to bind to CB1Rs as a negative modulator, and thus was considered an antagonist because it decreased the inward rectifying current that was produced by CB1 receptor activation [195]. Additionally, CBD’s bioavailability and plasma pattern through multiple routes of administration was similar to that of THC, meaning it was readily available at the receptors at around the same level as the CB1R partial agonist [180]. This is largely the reason why THC’s effects within the endocannabinoid system are largely attenuated by the presence of CBD. However, CBD could also exert effects within the expanded endocannabinoid system with direct receptor signaling at GPR55 as an antagonist, thus reducing excess presynaptic glutamate release, transient receptor potential cation channel subfamily V member 1 (TRPV1) as a negative modulator that works to block pain signaling, serotonin receptor 1A (5-HT1A) as an agonist exerting anxiolytic effects, and peroxisome proliferator-activated receptor-(PPAR) as an agonist that stimulates hippocampal neurogenesis [196,197,198,199,200]. Through the activation of these receptor-independent pathways, CBD directly resulted in intracellular effects such as Ca^2+^ homeostasis, AEA reuptake, and FAAH inhibition [115].

There seems to be promising literature that supports CBD as a possible therapeutic agent to be used for many different problems, including obesity (Table 4), anxiety, psychosis, and pain. In addition, the outcome that CBD has in mediating negatively produced THC effects could be used as a possible pathway in the future. However, more research must be done in order to fully understand the usefulness of this phytocannabinoid in certain treatments; specifically, how certain doses may respond along with coadministration of THC.

### General Effects of Cannabidiol (CBD) on Obesity Risks

There has not been much research published on the specific effects that CBD has on obesity risks in general. In what is available, the majority of work that was done was within the adult demographic (Table 4), and many of these studies agreed in that CBD administration resulted in many beneficial effects and could be used as a potential therapeutic agent for the treatment of obesity and diabetes complications [201]. Nevertheless, in the literature that was present for adolescent preclinical rodent testing, it was found that chronic administration of CBD within adolescents resulted in decreased levels of insulin resistance and improved the oxidative metabolism of glucose, an important process for restoring glycogen depletion [186]. In contrast, the authors of a study of prenatally exposed Sprague-Dawley rats concluded that the administration of CBD had no effect on the body weight of the rodent offspring, but did have an anti-inflammatory effect on the intestinal walls in a gastroschisis model [187]. Among adults, some studies, including [197], determined that CBD administration resulted in an upregulation of PPARγ activity. This was significant due to the role that the PPARγ receptor played in decreasing hepatic glucose levels and regulating lipid metabolism [202]. Additionally, with several studies confirming the therapeutic properties that CBD had as an anti-inflammatory and antioxidant agent [203,204], increasing efforts should be made in order to determine the efficacy of this drug not only as a neuroprotective agent, but as a potential mediator of obesity.

**Figure 2:**
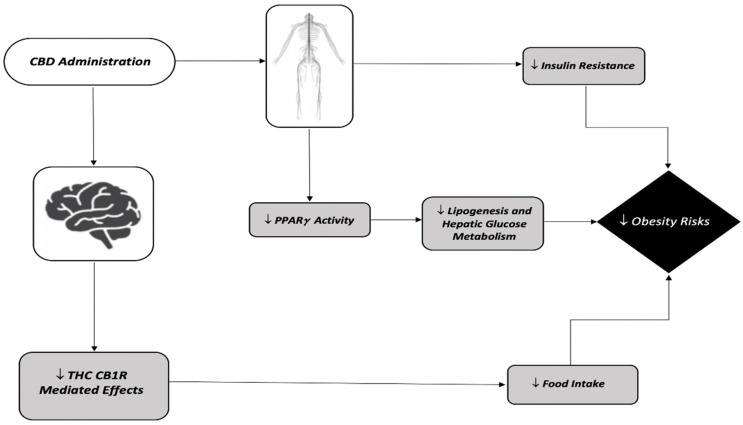
The mechanisms by which CBD administration affects the physiological responses to obesity risks through both the central and peripheral nervous systems. The central nervous system includes CBD inhibiting the THC-mediated CB1R effects that the drug has on obesity, specifically on food intake. The peripheral nervous system involves effects on insulin resistance along with PPAR-related activity that impacts hepatic glucose metabolism and lipogenesis levels within the adipose tissues. Symbols ↑ and ↓ denote an observed increase and decrease, respectively.

Currently, it is understood that CBD’s actions at the CB2Rs along with the negative modulation of the CB1Rs, and thus attenuation of THC’s effects (Figure 2), can be examined as a possible therapeutic mechanism in order to decrease obesity prevalence in society. Within the few studies that observed the direct effects of CBD on body weight and food intake, a great bulk of them observed a hypophagic effect along with a decrease in body weight among adults (Table 4). For instance, within a study measuring the appetitive effects that CBD had on THC-induced human subjects, it was found that CBD attenuated the hyperphagic response demonstrated by THC administration [193]. Similar results were observed within male Wistar rats, as CBD administration at 20 mg/kg attenuated the response of the CB1R agonist WIN55,212-22, which increased the overall food intake of the rats [188]. This reduction in overall food intake was thought to be CB1R-mediated, as CBD was acting as a negative modulator at this site within hypothalamic cells that were responsible for feeding behaviors [115,205]. However, the researchers in [206] proposed the role of CBD as a cytochrome P450 inhibitor. This would decrease the amount of THC being metabolized to 11-OH-THC, thus decreasing the amount of the active metabolite within the system. When not administered alongside a CB1R agonist, many studies saw an overall decrease in food intake. For example, in [194], a rodent study examining the effects of CBD administered at 10 mg/kg to male C57BL/6 mice, an overall effect of a decrease in food intake was seen. Furthermore, studies involving the administration of CBD within male rats found similar results with regard to food intake [115,191], with others observing a significant decrease in sucrose self-administration as well [192]. Not only has CBD been seen to affect food intake, but the authors of [189] suggested that chronic administration of a 2.5 or 5 mg/kg dose of CBD resulted in a significant decrease in body weight within a group of male Wistar rats. While the mechanism of the CB2Rs’ responsibility in regulating body weight is not well understood, the researchers in [207] explained CB2Rs as having a role in improving both glucose tolerance and metabolism. Within a cohort examining CBD’s effects on middle-aged rats with diabetes, chronic exposure was observed to not only decrease the overall body weight, but also to reduce diabetic factors that were linked to obesity [190].

Though much of the literature found supported CBD as a potential antiobesity agent, there were a few in which CBD had either no effect or the opposite effect on factors such as food intake and body weight (Table 4). For example, a study that observed the effects that CBD had on the negative consequences for diabetic rats determined that treatment with this drug had no effect on either body weight or blood glucose levels [203]. The researchers in [188] also found that systemic CBD administration with no CB1R agonist resulted in no difference in food intake, while those in [191] even saw an increase in body weight. This was explained by CBD administration causing an overall drop in energy expenditure within the rats [191].

In summary, the overarching effects of CBD are still yet to be fully understood, especially in the context of its role in regulating obesity. Further testing must be done in order to fully understand the mechanisms by which CBD attenuates food intake through the CB1R, and by which the drug decreases body weight through activation of the CB2R. This can allow for CBD to be unlocked as a potential therapeutic agent for not only neurological and inflammatory problems, but obesity-related issues in the future.

## 7. Conclusions

Since the times in which the plant *Cannabis sativa* was consumed among the people of ancient China, major advancements have been made in the understanding of phytocannabinoids and how each can result in different physiological responses when ingested. However, there are still many important questions that must be answered, specifically those regarding THC and how this compound affects the chances of obtaining obesity-related issues. Among adults and adolescents, there seemed to be a large portion of studies that observed THC as an agent that promoted factors such as increased food intake, body weight, and adipose tissue [139,152,153]. However, there were some data that suggested the opposite, which signified a need to study characteristics such as dose, regimen, and the route of administration.

The most important information in this review can be broken down further into the risks that cannabis smoking may have on obesity outcomes at different timepoints in life. Among adults and adolescents, administration of THC differentially affects obesity risks, and should therefore be further researched. However, with further data reporting an increased perception in society that regular cannabis smoking causes little to no harm, thus playing a large role in the increased use of this drug among pregnant women [165,166,167], there is increased cause for concern. This is mainly due to the strong relationship that prenatal smoking has on the birth weight outcomes of children. Cannabis administration throughout pregnancy was seen to have a direct effect by decreasing the birth weight, head circumference, and even different growth factors [173,174,175]. Proposed mechanisms consisted largely of fetal hypoxia from smoke inhalation and inhibition of the endothelial growth factor from CB1R activation [172,179]. This lack of fetal growth can have great implications for many negative outcomes that may arise later in a child’s adolescence, such as an increased risk of obesity-related issues. Further research is still needed in order to fully understand the ramifications that cannabis may have on our society in general. With the increasing rate of legalization of recreational use of this plant, there is a greater need to fully understand both the therapeutic and adverse aspects that these phytocannabinoids may provide.

## Figures and Tables

**Table 1:** A summary of preclinical and clinical studies in adults treated with THC and outcomes on obesity-related symptoms. Most symptoms included were based on food/caloric intake, body weight, and BMI, but some other obesity-related features included energy expenditure, insulin resistance, and visceral fat content. The limited information in certain sources resulted in some of the information that would have been contained within the table being omitted.

Species	Regimen	Sex	THC Dose	Route of Administration	Obesity Risk Outcomes	Reference
Rat	Acute	Male	0.5, 1.0, 2.0, or 4.0 mg/kg	Oral	↑ food intake, ↓ eating latency	[112]
Rat	Acute	Male	0.5, 1.0, 2.0, or 4.0 mg/kg	Oral	↑ food intake, ↓ eating latency	[120]
Rat	Chronic (30 straight days)	Male	4.0, 8.0 mg/kg	Intraperitoneally (4.0 mg/kg), Orally (8.0 mg/kg)	↓ body weight, ↓ food intake	[144]
Rat	Acute	Male	0.5, 1.0, 2.5 mg/kg	Intraperitoneally	↑ food intake in all diets, ↑ sucrose palatability	[121]
Rat	Acute	Male	2.5, 5.0 mg/kg	Subcutaneous	↓ energy expenditure	[145]
Rat	Acute	Male	2.5 mg/kg	Intraperitonially	↑ sucrose palatability, ↑ ingestive responding	[122]
Human	Chronic (survey)	Male and female	Self-report	Smoked	↓ obesity risks	[142]
Human	Chronic (meta-analysis)	Male and female	Self-report (users and nonusers)	Smoked	↑ caloric intake, ↓ body weight, ↓ obesity	[143]
Human	Chronic (survey)	Male and female	Self-report (over 15 years: never used, <180 days, 180–1799 days, ≥1800 days)	Smoked	↑ appetite, ↑ caloric intake, ↑ body weight, ↑ waist size	[152]
Human	Acute	Male and female	2.0%, 3.9%	Smoked	↑ food intake, ↑ body weight at high doses, ↑ caloric intake from fat	[153]
Human	Chronic (survey)	Male and female	Self-report (nonusers and users)	Smoked	↓ levels of BMI, ↓ % fat mass, ↓ fasting insulin, ↓ insulin resistance	[154]
Human	Chronic (survey)	Male and female	Self-report (at least 4 days a week for the past 6 months)	Smoked	↑ carbohydrate intake, ↑ visceral fat tissue, ↑ blood pressure, ↑ adipose tissue insulin resistance, ↑ leptin and ghrelin, ↓ PYY	[139]
Human	Acute	Male and female	50.6 mg	Oral, smoked, vaporized	↓ blood insulin concentrations, ↓ GLP-1 levels	[133]
Human	Chronic (survey)	Male and female	Self-report	Smoked	↓ waist circumference, ↓ fasting insulin, ↓ insulin resistance, ↓ HDL-C	[155]
Human	Chronic (survey)	Male and female	Self-report (never, former, current use)	Smoked	↑ rate of prediabetes	[156]
Mice	Acute	Male	1.0, 3.0, 10.0, 30.0, 56.0 mg/kg	Intraperitoneally	↑ caloric intake	[127]
Mice	Acute	Cell cultures	1 mg/kg	N/A	↓ in fat content, ↑ in IRS-1/2, ↑ GLUT4	[146]

Note: ↑ = Observed increase, ↓ = Observed decrease, BMI = Body mass index, PYY = Peptide YY, GLP-1 = Glucagon-like peptide 1, HDL-C = High-density lipoprotein cholesterol, IRS-1/2 = Insulin receptor substrate 1/2, GLUT4 = Glucose transporter type 4, N/A = Not available.

**Table 2:** A summary of preclinical and clinical studies in adolescent subjects treated with THC and the primary outcome that it had on inducing obesity-related symptoms. Included are an array of different obesity risk outcomes from THC use, including food intake, body weight, plasma insulin, and glucose uptake. The limited information in certain sources resulted in some of the information that would have been contained within the table being omitted.

Species	Regimen	Sex	THC Dose	Route of Administration	Obesity Risk Outcomes	Reference
Rats	Acute	Male	0.5, 1.0, 2.0 mg/kg	Oral	↑ total food intake, ↓ eating latency, ↑ total duration of eating	[112]
Rats	Acute	Female	0.1, 0.5, 2.0 mg/kg	Intraperitoneally	↑ total food intake, ↓ body weight loss, ↓ energy expenditure	[161]
Rat	Chronic (16 straight days)	Male	3, 5, 6, 8, 10 mg/kg	Subcutaneously, Oral	↓ adiposity, ↓ plasma insulin	[131]
Rat	Acute	Male	0.01, 0.05, 0.1, 0.5, 1.0 mg/kg	Intravenously	↑ glucose uptake at lower concentrations, ↓ glucose uptake at high blood THC levels	[116]
Human	Chronic	Male and Female	Self-report (ranged from never to 40 times or more within a given month)	Smoked	↑ proportion of being overweight, ↑ likelihood of obesity	[160]
Mice	Chronic (28 straight days)	Male	2, 4 mg/kg	Intraperitonially	↓ weight gain in DIO mice, ↓ energy intake, ↓ fat mass	[119]
Mice	Acute	Male	10 mg/kg	Intraperitonially	↓ glucose uptake, ↓ glycolysis, ↓ lactate release	[135]
Large white pig	Acute	Male	0.05, 0.1, 0.2 mg/kg	Intravenously	↑ levels of THC in fat tissues, ↑ time in fat tissues	[141]

Note: ↑ = Observed increase, ↓ = Observed decrease, DIO = Diet-Induced Obese.

**Table 3:** A summary of preclinical and clinical trials that represent prenatal THC administration and the primary outcome that it had on inducing postnatal issues that can eventually lead to increased obesity risks later into the child’s adolescence, and even later in life. A majority of the obesity risk outcomes from the administration of THC were based on birth weight, but also levels of fetal stress during pregnancy. The limited information in certain sources resulted in some of the information that would have been contained within the table being omitted.

Species	Regimen	Sex (Child)	THC Dose	Route of Administration	Obesity Risk Outcomes	References
Rat	Chronic (15.5 straight days)	Male and female	3 mg/kg	Intraperitoneally	↓ fetal growth, ↓ expression of GLUT1, ↑ intrauterine growth restriction	[171]
Human	Chronic (multiple studies, no specific regimen)	Male and female	Self-report	Smoked	↑ overweight children, ↑ obesity risks	[172]
Human	Chronic (substance use through pregnancy)	Male and female	Self-report (exposure before or after knowledge)	Smoked	↓ birth weight, ↓ intracranial volume, ↓ white matter volume	[173]
Human	Chronic	Male and female	Self-report (during pregnancy, ever regular, lifetime)	Smoked	↓ birth weight, ↑ preterm birth, ↑ admission to NICU	[174]
Human	Chronic (8 straight days)	Cell culture	0.5–1.5 mg	Intratumorally	↓ levels of phosphorylated VEGFR-2, ↓ endothelial growth factor expression	[175]
Mice	Chronic (12 straight days)	Male and female	200 mg cigarettes	Smoked	↓ birth weight	[176]

Note: ↑ = Observed increase, ↓ = Observed decrease, GLUT1 = Glucose transporter type 1, NICU = Newborn intensive care unit, VEGFR-2 = Vascular endothelial growth factor receptor 2.

**Table 4:** An analysis of both preclinical and clinical trials that represent CBD administration and the primary outcome it had on inducing obesity-related symptoms. It should be noted that the majority of papers were based on the adult demographic, with the exception of the first two references, which were based on adolescent and prenatal age groups, respectively.

Species	Regimen	Sex	CBD Dose	Route of Administration	Obesity Risk Outcomes	References
Rats	Chronic (14 consecutive days of exposure)	Male	10 mg/kg	Intraperitoneally	↓ insulin resistance, ↑ oxidative metabolism of glucose	[186]
Rats	Acute	Male and female	30 mg/kg	Intraperitoneally	↔ body weight	[187]
Rats	Acute	Male	1, 10, 20 mg/kg	Intraperitoneally	↓ hyperphagia with CB1 agonist, ↔ food intake	[188]
Rats	Chronic (14 consecutive days of exposure)	Male	2.5, 5 mg/kg	Intraperitoneally	↓ body weight	[189]
Rats	Chronic (30 consecutive days of exposure)	Male	10 mg/kg	Intraperitoneally	↓ body weight, ↓ diabetic outcomes	[190]
Rat	Acute	Male	3 mg/kg	Intraperitoneally	↓ food intake, ↑ body weight	[191]
Rats	Acute	Male	0.044, 0.44, 4.4 mg/kg	Orally, subcutaneously	↓ food intake	[115]
Rats and mice	Chronic (24 consecutive days of exposure)	Male	20, 40 mg/kg	Intraperitoneally	↓ sucrose administration	[192]
Human	Acute	Male and female	High CBD: low THC; low CBD: high THC	Smoked	↓ food intake, ↓ hyperphagia	[193]
Mice	Acute	Male	10 mg/kg	Intraperitoneally	↓ food intake	[194]

Note: CBD = Cannabidiol, ↑ = Observed increase, ↓ = Observed decrease, ↔ = No observed change.

## Data Availability

No new data were created or analyzed in this study. Data sharing is not applicable to this article.
